# Enhancing the insecticidal activity of new *Bacillus thuringiensis* X023 by copper ions

**DOI:** 10.1186/s12934-020-01452-8

**Published:** 2020-10-17

**Authors:** Zhuolin Liu, Junyan Xie, Ziru Deng, Mulan Wang, Dandan Dang, Sha Luo, Yunfeng Wang, Yunjun Sun, Liqiu Xia, Xuezhi Ding

**Affiliations:** Hunan Provincial Key Laboratory of Microbial Molecular Biology, State Key Laboratory of Developmental Biology of Freshwater Fish, College of Life Science, Hunan Normal University, Changsha, 410081 China

**Keywords:** *Bacillus thuringiensis*, Copper ion, ICP, Proteome, *Plutella xylostella*

## Abstract

**Background:**

A new *Bacillus thuringiensis* X023 (BtX023) with high insecticidal activity was isolated in Hunan Province, China. The addition of metals (Cu, Fe, Mg and Mn) to the medium could influence the formation of spores and/or insecticidal crystal proteins (ICPs). In previous studies, Cu ions considerably increased the synthesis of ICPs by enhancing the synthesis of poly-β-hydroxy butyrate. However, the present study could provide new insights into the function of Cu ions in ICPs.

**Results:**

Bioassay results showed that wild strain BtX023 exhibited high insecticidal activity against *Plutella xylostella*. The addition of 1 × 10^−5^ M Cu^2+^ could considerably increase the expression of *cry1Ac* and *vip3Aa*, and the insecticidal activity was enhanced. Quantitative real-time polymerase chain reaction (qRT-PCR) and proteomic analyses revealed that the upregulated proteins included amino acid synthesis, the glyoxylate pathway, oxidative phosphorylation, and poly-β-hydroxy butyrate synthesis. The Cu ions enhanced energy metabolism and primary amino acid synthesis, will providing abundant raw material accumulation for ICP synthesis.

**Conclusion:**

The new strain BtX023 exerted a strong insecticidal effect on *P. xylostella* by producing ICPs. The addition of 1 × 10^−5^ M Cu^2+^ in the medium could considerably enhance the expression of the *cry1Ac* and *vip3Aa* genes, thereby further increasing the toxicity of BtX023 to *Helicoverpa armigera* and *P. xylostella* by enhancing energy synthesis, the glyoxylate cycle, and branched-chain amino acids synthesis, but not poly-β-hydroxy butyrate synthesis.

## Introduction

Insecticidal crystal proteins (ICPs) produced by *Bacillus thuringiensis* (Bt) are well-known eco-friendly biological pesticides and insecticides with high specificity and efficiency. ICPs, except for the vegetative insecticidal protein (Vip), were formed during sporulation [1], and were also known as “parasporal crystalline” [2]. The *cry* gene of Bt is responsible for the insecticidal activity and has been successfully transferred to plants to provide resistance to pests [3–5]. However, insect resistance to Bt inevitably develops with the increase in the usage of bioinsecticides; thus, strategies to overcome this resistance are developing [6], such as isolating new serotypes strains and genetic engineering, are needed.

Several factors, including mineral element, affect ICP productivity. Metal ions, which are cofactors or electrolytes that balance the charge between the inner and outer membranes of the organism, serve important functions [7]. In *B. thuringiensis*, Mg^2+^ is related to the biosynthesis of 135 and 65 kDa toxic components; Ca^2+^, Fe^2+^ and Mn^2+^ are involved in the specific stimulation of several types of ICPs; Mn^2+^ in particular can promote the yield of spores [8–10]. The divalent metal ions affecting enzymatic functions show certain similarities, and *Bacillus* spp. [11, 12], particularly Bt [11–16], can absorb certain heavy metal ions, including Cu, Cr, Cd and Ni. Ni^2+^ elicits hazardous effects by activating many enzymes, such as alkaline phosphatase, oxaloacetate decarboxylase, and urease in certain *Bacillus* spp*.* (*B. pastuerii*, *B. subtilis*, and *B. sphaericus*) [17–19]. The previous study proposed that Cu^2+^ increases PhaR expression and consequently changes carbon flow; the increase in carbon sources, which are used to produce intracellular poly-β-hydroxybutyrate (PHB) as a storage material, lead to increased ICP production [16]. However, another report showed that the there is no direct association between the PHB accumulation and the sporulation and ICP formation in *B. thuringiensis* as carbon-energy storage via the deletion of *phaC* and *phaZ* [23]. Furthermore, the function of Cu^2+^ in ICP production is unclear.

A new Bt strain (X023) was isolated from the forest soil of Xiangtan City, Hunan Province, China. This area is close to an artificial lake that is rich in heavy metal ions. We added 1 × 10^−5^ M CuSO_4_ or 1 × 10^−6^ M NiSO_4_ (after concentration gradient screen) in the medium (Figure S1) to determine the difference between their effects. The insecticidal activity was improved only with the addition of Cu^2+^. Proteomics and qRT-PCR analyses were performed to explore the possible pathway by which metal ions affect ICP synthesis. This work provided an interesting perspective on the study of the Bt ICP synthetic pathway.

## Results

### Microscopic observation of BtX023

**Fig. 1:**
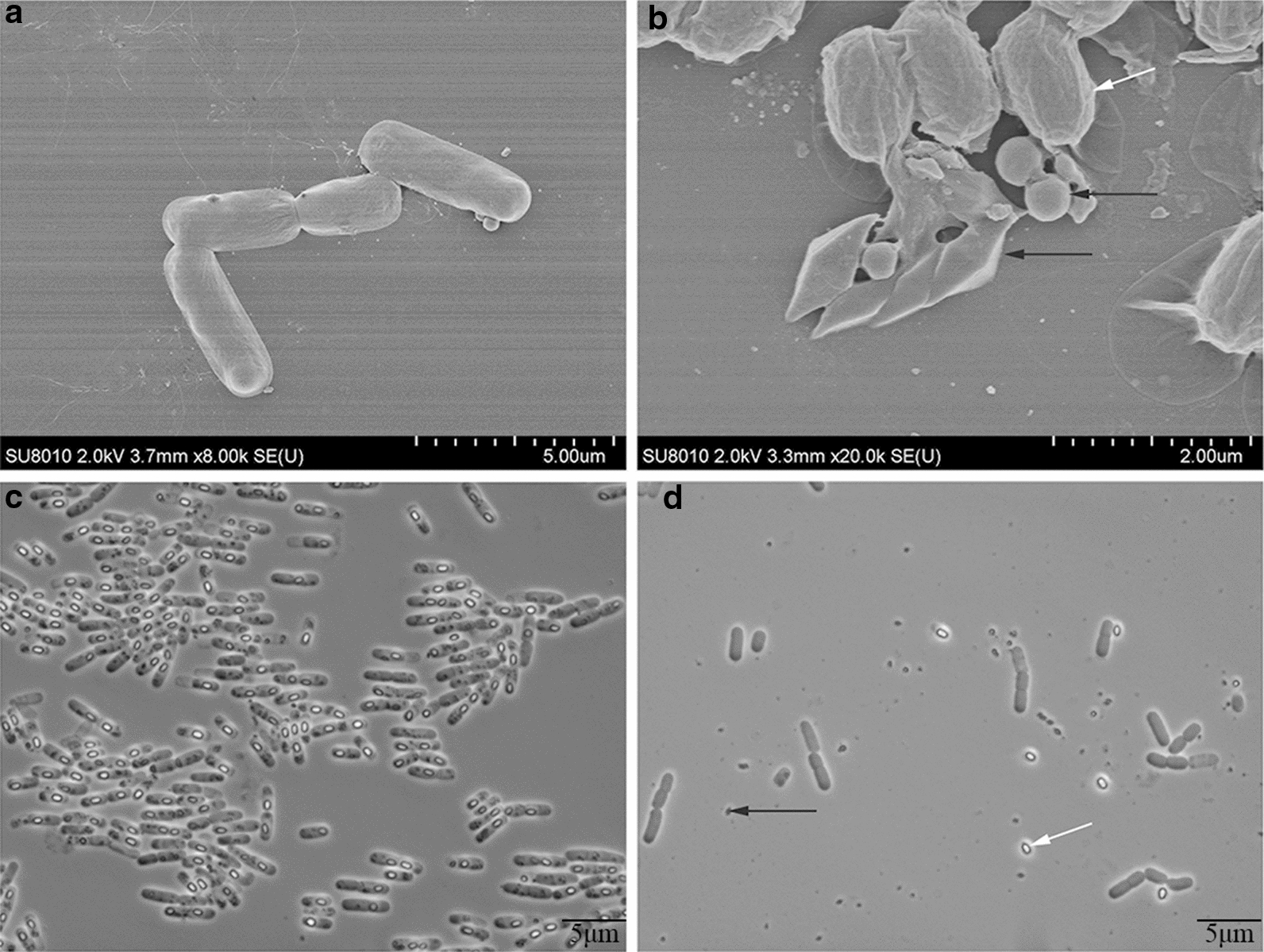
Microscopic observation of the BtX023 strain. **a** single cell morphology observed by electron microscopy (24 h); **b** electron microscopic observation of crystal protein and spores (36 h), black arrow indicates the rhomboid and spherical companion crystal, white arrow indicates the spores; **c**, **d**, Cell morphology after fermentation for 30 h and 48 h

The strain was elliptical at the stationary phase, and the size of the mother cells was 2.7–4.5 μm × 1.3–1.5 μm. The spores were 1.0–1.7 μm × 0.7–1.0 μm. The diamond-shaped crystal was 1.2–1.6 μm × 0.4–0.6 μm, and the spherical companion crystal had a diameter of 0.3–0.6 μm (Fig. 1a, b, c). After 60 h of fermentation, the samples were separately observed by phase-contrast microscopy and scanning electron microscopy. The cells were mostly lysed, and a large number of spores and parasporal crystals were released (Fig. 1d).

### 16S rRNA identification of a new BtX023

**Fig. 2:**
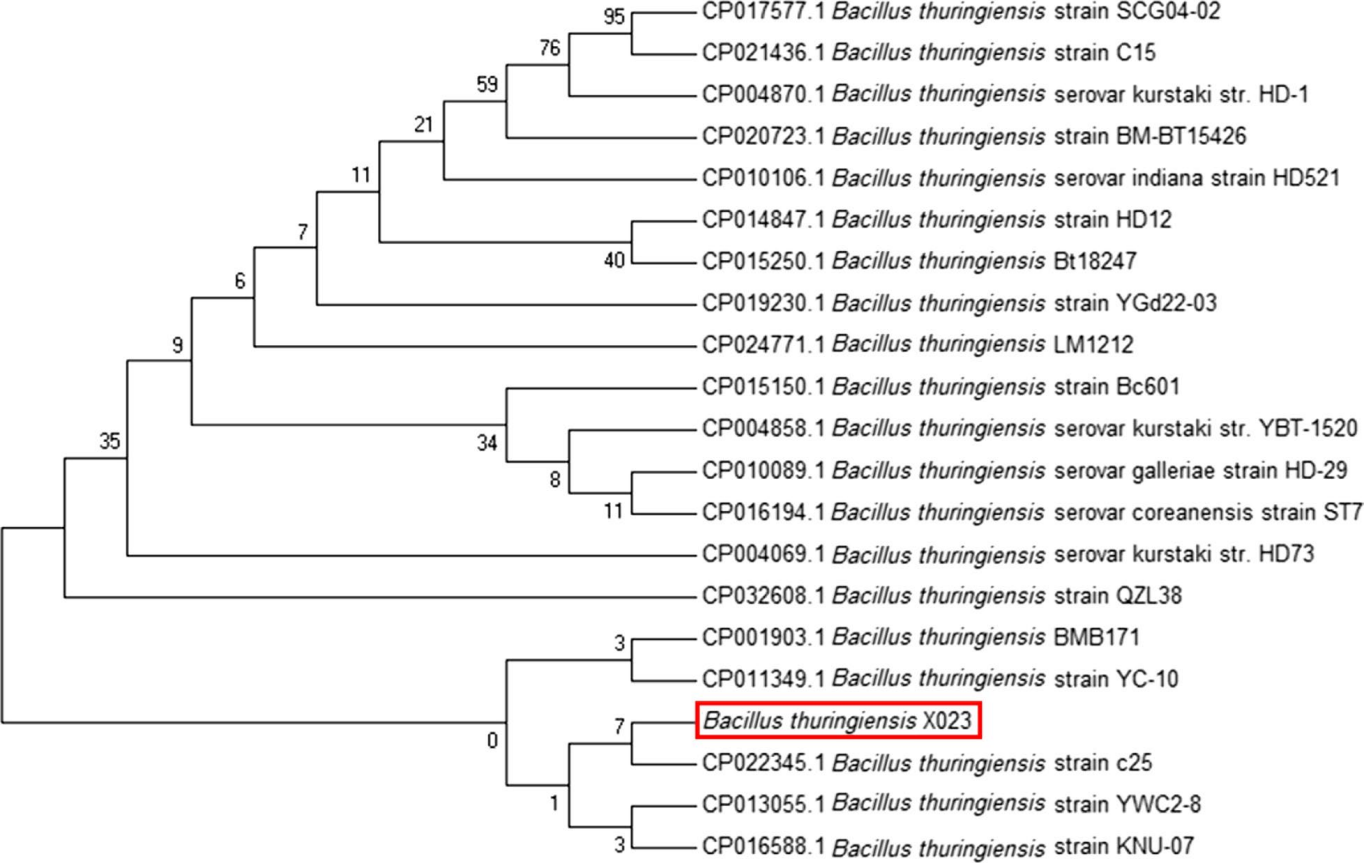
16S rRNA phylogenetic tree of BtX023. BtX023 is marked with red rectangle, which has high homology to several strains like Btc25, BtKUN-10, BtYWC2-8, etc

The genome was extracted, and the 16S rRNA of BtX023 was amplified and sequenced, followed by comparison analysis using the BLAST database. A phylogenetic tree (with the neighbor-joining method) was constructed. It showed the BtX023 with the highest homology to the Bt strain c25 (Fig. 2).

### Growth curve of BtX023 after adding Cu and Ni ions to the fermentation medium

**Fig. 3:**
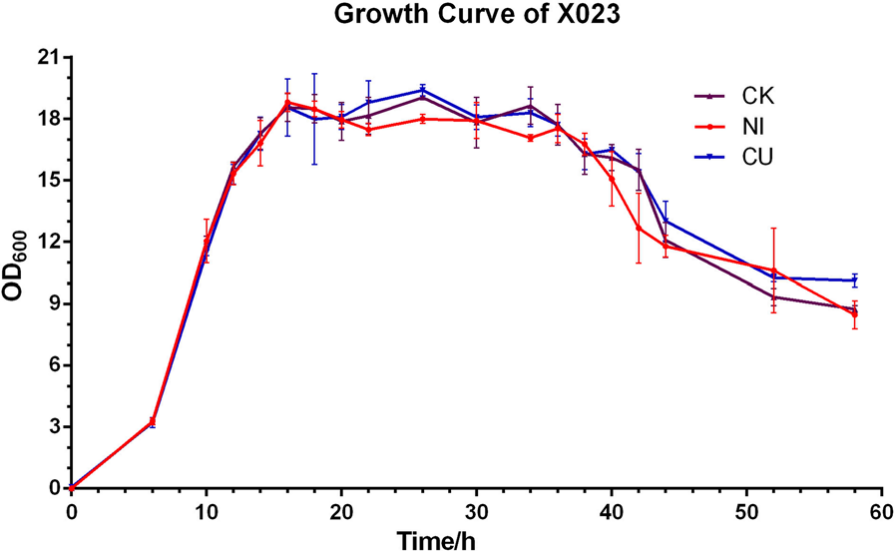
Growth curve of *Bacillus thuringiensis* X023 in different mediums. Black line is the CK without any addition for the original fermentation medium, blue line is the CU, which was added the Cu^2+^ at a final concentration of 1 × 10^–5^ M, red line is the NI that the Ni^2+^ at a final concentration of 1 × 10^–6^ M

The media were mixed with 1 × 10^−5^ M CuSO_4_ (CU) and 1 × 10^–6^ M NiSO_4_ (NI). The original fermentation medium was used as the CK. Three biological replicates were set for each medium when measuring the growth curve. Samples were minored every 2 h to plot the growth curve (Fig. 3). The media with three treatment did not significantly affect the growth and the duration parameters of each growth period. The lag phase was the initial 2 h, and the logarithmic growth phase was 2–16 h after fermentation, entering the decline phase at 36 h.

### Effect of Cu ions on the expression of ICPs and insecticidal activity

**Fig. 4:**
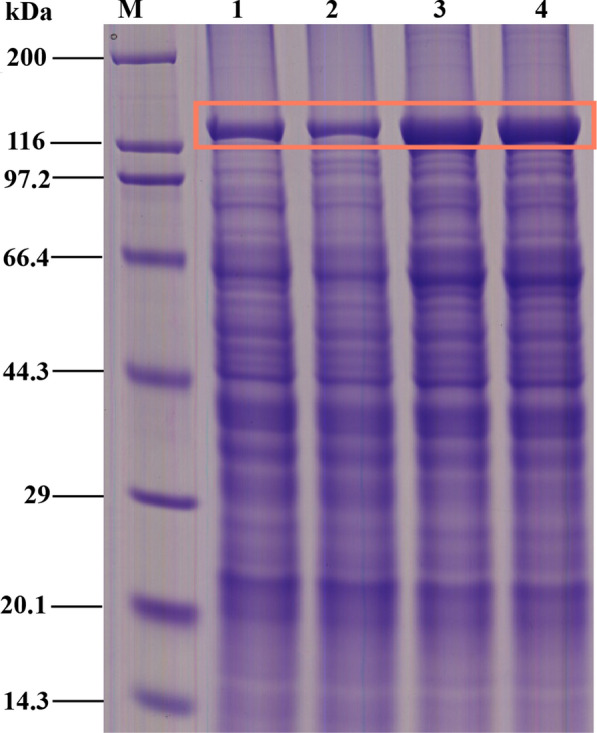
SDS-PAGE of total proteins of the 36 h. M, Protein Marker; 1–2, CK; 3–4, CU

We extracted the whole-cell proteins at 36 h. SDS-PAGE was performed on the samples with 10 μg of protein (Fig. 4). Based on the gray scale analysis of the strip with the Gel-Pro analyzer 4.0, CU had stronger 130 kDa band (the IDOs of lines 3 and 4 were 202.08 and 191.10, respectively) than that of CK (the IDOs of lines 1 and 2 were 76.679 and 61.841), which CU was 2.8 fold of CK. Remarkable changes were observed in the overall protein expression levels of different media treatments. In particular, the 130 kDa protein bands differed significantly. Two kinds of agricultural lepidopteran pests were used (Table 1). The insecticidal activity of BtX023 strain against *Plutella xylostella* and *Helicoverpa armigera* were increased with 65% and 35%, respectively.

**Table 1:** LC_50_ analysis of *Bacillus thuringiensis* X023 fermentation broth from three media

Sample	LC_50_ (μL/mL) to *P. xylostella*	95% CI	LC_50_ (μL/mL) to *H. armigera*	95% CI
CK	2.462	1.642−3.822	6.179	4.395−9.593
CU	1.489	1.358−1.642	4.569	3.781−5.389
Cu^2+^ sup	_	_	_	_

95% CI, 95% confidence intervals, non-overlapping 95% confidence intervals of LC_50_ were used as the criteria to determine significant difference in toxicities among different treatments

*CK* fermentation broth from the original medium, *CU* fermentation broth from 1 × 10^-5^ M Cu^2+^-added medium, *Cu*^*2+*^* sup* the supernatant of CU, ^__^ without toxicity

### Proteomic analysis

Label-free quantification (LFQ) proteomic detected 17,493 peptides. The number of unique peptides was 10,668, and the number of proteins identified was 1818. A total of 1532 proteins were obtained by GO annotation (https://www.geneontology.org). In addition, 1702 proteins were obtained by eggNOG (https://eggnogdb.embl.de/), and 1417 proteins were obtained by KEGG annotation (https://www.kegg.jp/). The mass of these proteins ranged from 10 to 60 kDa (Additional file 1). Moreover, the ICP data showed that BtX023 could produce three types of ICPs, namely, Cry1, Cry2Aa, and Vip3Aa. The most common ICP was Cry1. Cry1A was the most significant among the Cry1 proteins, particularly Cry1Ac. The other Cry1 proteins, such as Cry1I, Cry1F, and Cry1E (Additional file 2), were not discussed because of their low amounts, although they also have high specific toxicity to Lepidopteran.

**Fig. 5:**
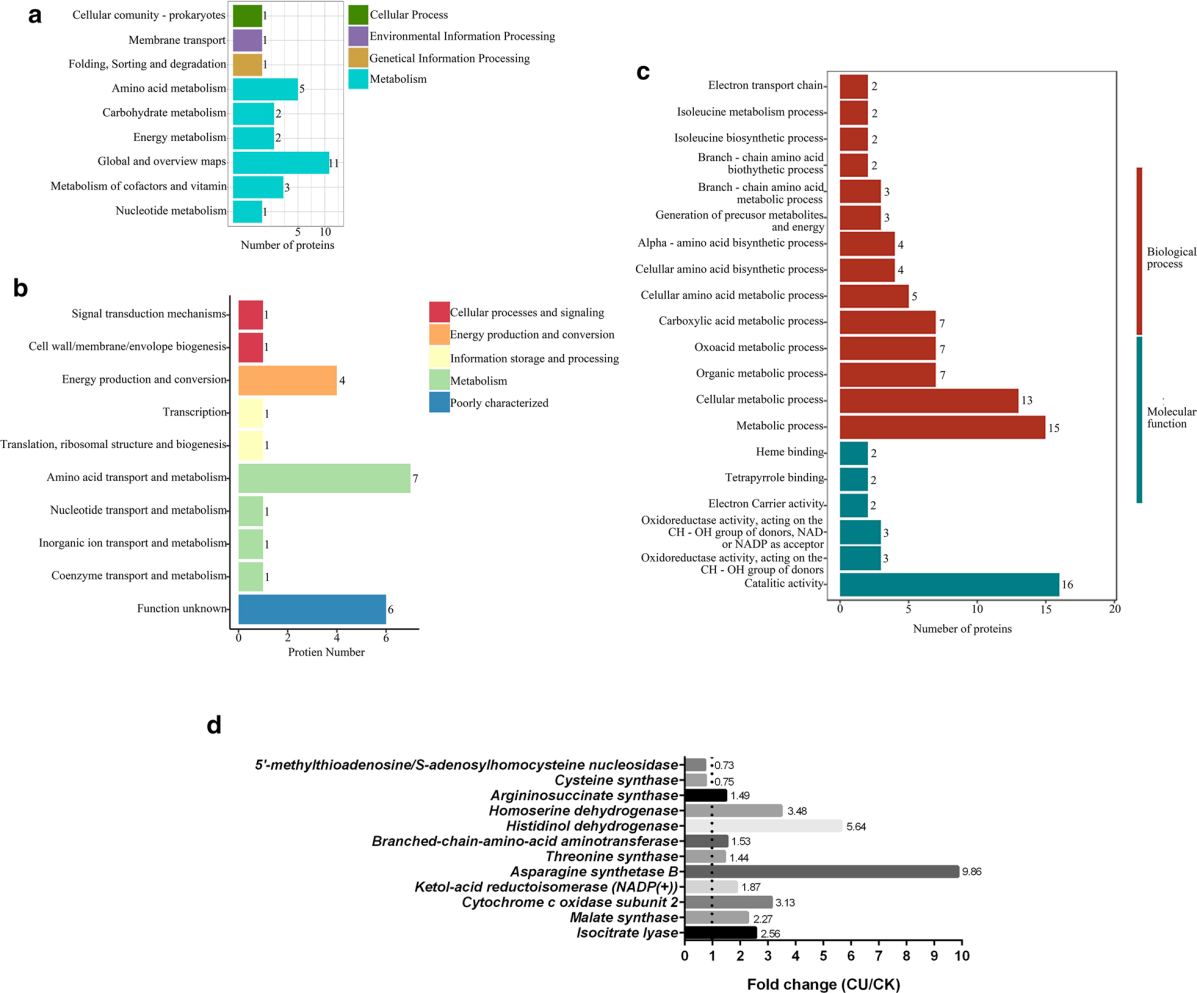
Differential proteomic analysis which CU compared to CK. **a** KEGG analysis, biological pathways involved in differential proteins. The abscissa is the number of differential proteins, and the ordinate is the main biological pathway. **b** eggNOG analysis, the abscissa is the number of differential proteins, and the ordinate is the main protein category. **c** GO analysis of the differential proteins. **d** Fold-change of major differential proteins

Differential proteomic analysis was carried out between CU and CK (Additional file 3). A total of 1329 proteins were quantified, of which 27 were upregulated and 19 were downregulated. Statistical analysis of differential proteins of KEGG, GO, and eggNOG (Fig. 5) showed that the addition of Cu^2+^ mainly affected energy metabolism and amino acid synthesis. Quantitative analysis of differential proteins revealed that the expressions of the oxidase II subunit of cytochrome c (3.13-fold), isocitrate lyase (2.56-fold), and malate synthase (2.27-fold) were significantly upregulated. In addition, the enzymes related to the synthesis of branched-chain amino acids (BCAAs) leucine, isoleucine, and valine were upregulated. Ketol-acid reductoisomerase, BCAA aminotransferase, and Val-tRNA ligase were upregulated by 1.87-, 1.53-, and 1.40-fold, respectively, compared with CK. For other amino acids, the histidinol dehydrogenase, homoserine dehydrogenase, and asparagine synthetase B were upregulated by 5.64-, 3.48-, and 9.86-fold, respectively, compared with CK. However, cysteine synthase and 5′-methylthioadenosine are downregulated by 0.75- and 0.73-fold, respectively, compared with CK.

### qRT-PCR analysis of some critical genes

**Fig. 6:**
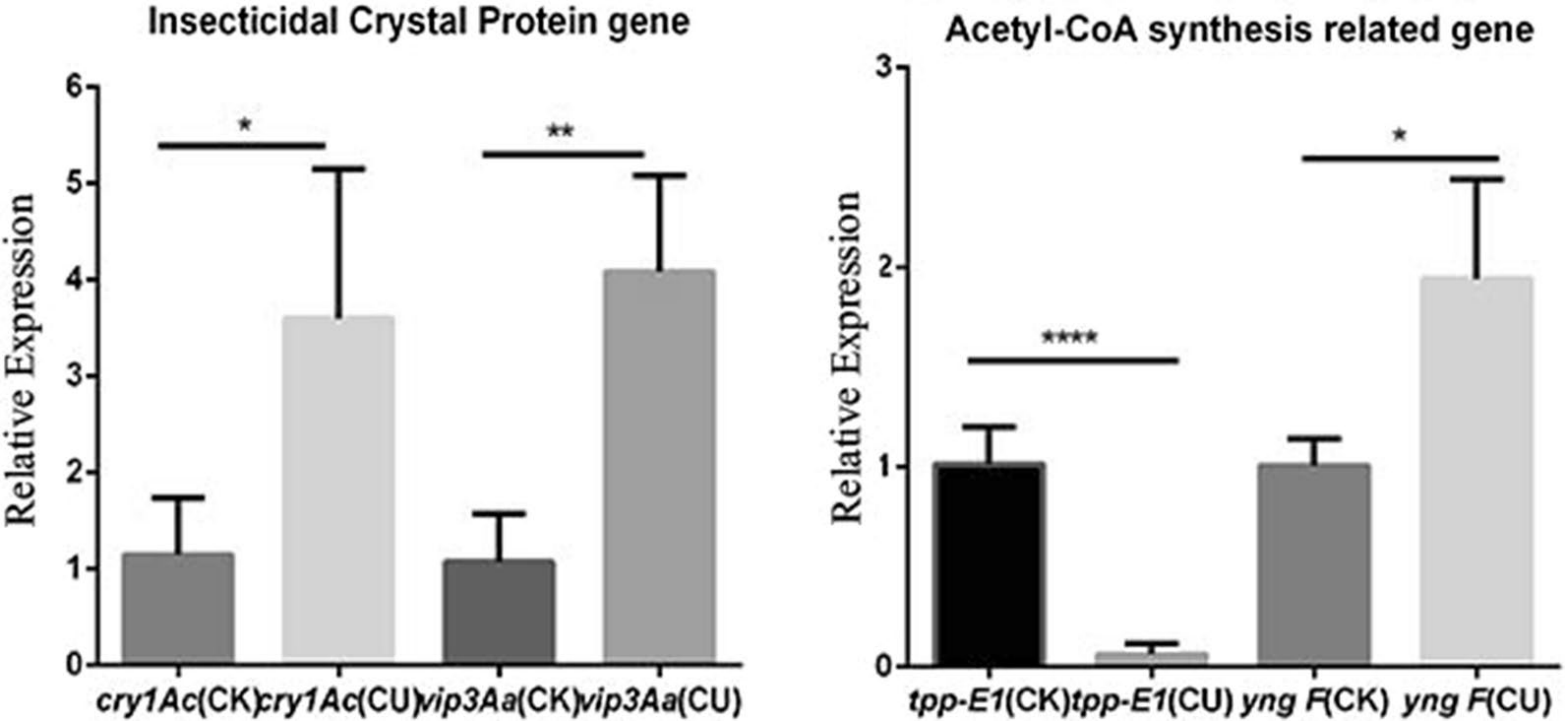
Transcription level of two insecticidal proteins and two acetyl-CoA synthesis-related proteins. Two-tailed T test, * p < 0.05, ** p < 0.005, **** p < 0.0001

Some of the above-mentioned key proteins were also verified by qRT-PCR. The fold change of the *ilvC*, *ilvE*, *coxB*, *aceA*, and *aceB* transcription level was consistent with their above-mentioned protein levels (Fig. 5d). However, the transcription levels of the *cry1Ac* and *vip3Aa* of CU were more than threefold that of CK. *yngF* and *tpp-E1*, which were associated with the production of acetyl-CoA, were also tested with qRT-PCR. The transcription level of the *yngF* was upregulated in CU, whereas that of the *tpp-E1* was downregulated (Fig. 6).

## Discussion

Cu^2+^ can increase the expression level of insecticidal proteins of BtX023, and the enhanced toxicity to pests is similar to previous reports [16, 20]. Previous reports indicated that Cu^2+^ ions considerably affect the synthesis of PHB but does not significantly improve it. In the present study, the main influence of Cu ions is on the energy and the synthesis of BCAAs.

The pathway of energy metabolism that was affected by Cu^2+^ was the glyoxylate cycle. *aceA* and *aceB* were upregulated, which involved in the glyoxylate cycle. This cycle, as a metabolic bypass of the TCA cycle, can utilize acetyl CoA more efficiently than the TCA cycle to provide more ATP feedstock for oxidative phosphorylation [21]. In another study, Cu^2+^ is a key component of the cytochrome c oxidase II subunit, which allowed a large number of electrons to combine with oxygen molecules, whereas a lot of protons were pumped from the matrix to the periplasmic space. The high concentration gradient of the proton concentration difference can form a large amount of ATP. The above-mentioned finding can be contacted by energy flow.

Furthermore, the downregulated TppE1 protein can decrease the pyruvate and convert into acetyl-CoA, and then, the acetyl-CoA flow is reduced in the citric acid cycle. Conversely, this process may allow more pyruvate to flow into other pathways, such as in amino acid synthesis, particularly the BCAAs, which are required for ICPs.

The amino acid metabolism is affected after adding Cu^2+^ into the medium for *B. thuringiensis*. BCAAs are an important part of the amino acid composition of ICPs in Bt [22]. From the results of analyses of different proteins and qRT-PCR, we observed that the expression levels of *ilvC* and *ilvE* were upregulated. The *ilvC* and *ilvE* were involved in the synthesis of BCAA (Leu, Val and Ile) from pyruvate. For other kinds of amino acids, the proteins homoserine dehydrogenase (Hom), threonine synthase (ThrC), and AspB were upregulated in the CU, which was related to the conversion of asparatate to homoserine, threonine, and asparagine. In addition, for the differential proteins, the glutamate synthase and arginine succinate synthase were slightly upregulated. However, the enzymes involved in the synthesis of methionine and cysteine (CysK and MtnN, respectively) were slightly downregulated, probably because CysK is directly involved in the synthesis of cysteine, and MtnN is involved in the conversion of methionine to cysteine [23]. Furthermore, cysteine is a non-essential amino acid, which could combine with Cu ions to form insoluble thiolates.

The raw materials of amino acids required for the synthesis of ICPs are also derived from the hydrolysis of proteins [24]. The proteomic results showed that many proteases, such as oligopeptidase F (YngF) and trypsin-activity protein (W8YX33), were significantly upregulated. However, the expression of peptidase M20 (an aminopeptidase, A0A243G153) was significantly reduced, and the downregulation was 0.28-fold. The peptidase M20 primarily cleaved the zinc metalloprotease, which is a compound that can improve the formation of ICP.

**Fig. 7:**
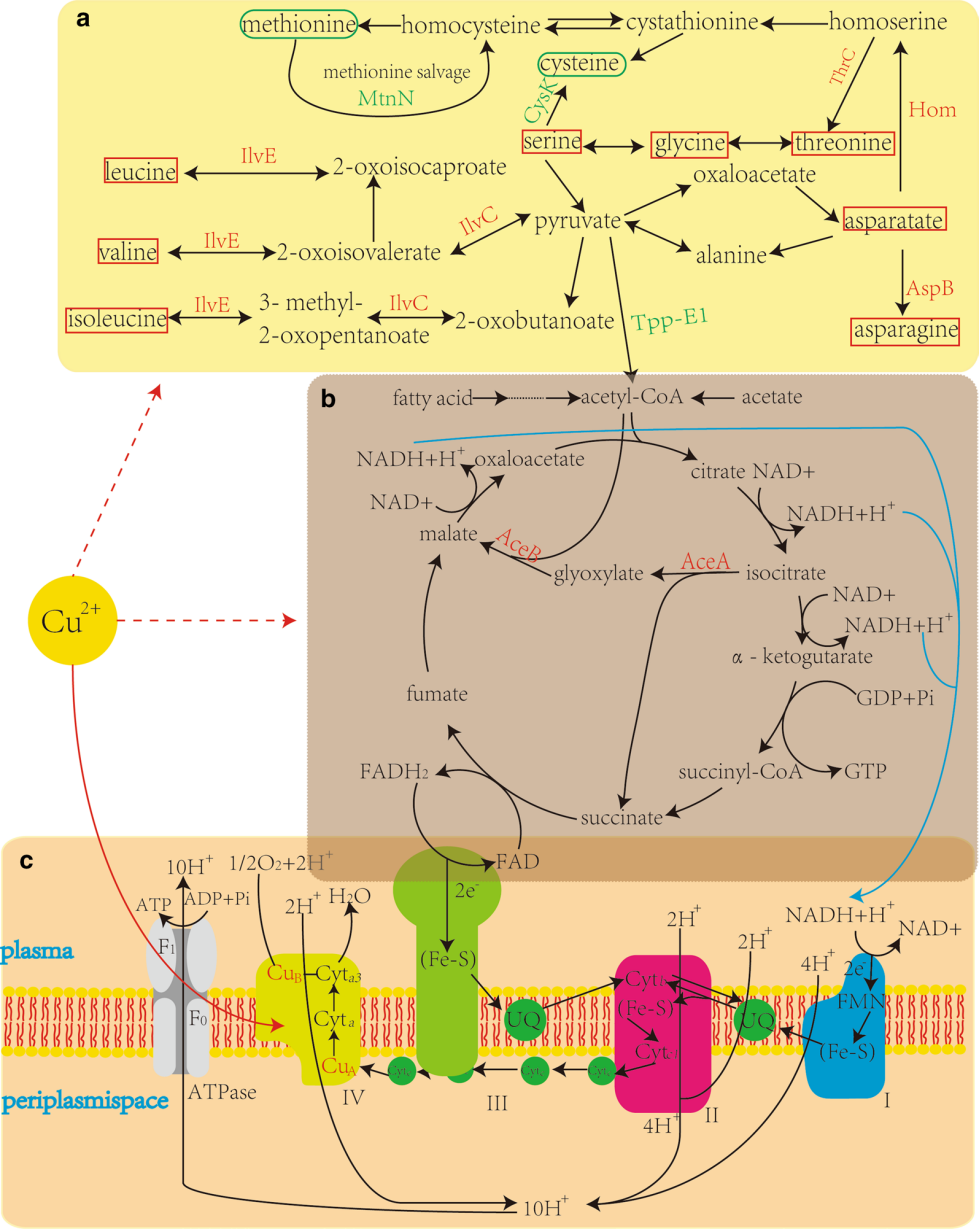
The diagram of metabolic pathways may be affected by addition of copper ions for BtX023. The solid red line indicates direct effect, and the red dotted line indicates indirect effect. The proteins marked in red are up-regulated, and the green are down-regulated. The blue line represents the NADPH^+^ produced from the Krebs cycle into the respiratory chain. **a** Synthesis of BCAA and some other amino acids. The red boxes are labeled with amino acids that may be synthetically upregulated, and the green rounded boxes are labeled with amino acids that may be downregulated. **b** Citrate cycle and oxaloacetate cycle pathway, and the expression of both key proteins are significantly upregulated. **c** Oxidative phosphorylation in which the portion of the IV subunit (cytochrome c oxidase) containing Cu^2+^ is indicated in red

We proposed that the addition of Cu^2+^ could upregulate the expression level of the key components of the respiratory chain and indirectly influence the glyoxylate cycle and BCAA synthesis, thereby further enhancing energy metabolism and amino acid synthesis and providing abundant raw material accumulation for ICP synthesis (Fig. 7). The production cost of Bt preparation is reduced, and a new technical approach is provided for the research and development of environmental protection and high efficiency Bt preparation. And the new mechanism of metabolic regulation of Cu^2+^ to enhance the synthesis of insecticidal crystalline proteins was revealed, which has important scientific significance and application value.

## Conclusion

This research found a new strain BtX023. The addition of Cu^2+^ improved the insecticidal toxicity of BtX023 against *P. xylostella* and *H. armigera* by increasing the expression of Cry and Vip3A proteins. The effectiveness of Cu^2+^ was related to its effects on energy and amino acid metabolism. The electron transport chain, the glyoxylate cycle, and BCAA synthesis pathway could influence and change the metabolic flux for the formation of ICPs, but not PHB. The optimization of fermentation increased the ICP yield and provided a scientific basis for its industrial application. In addition, such optimization had important practical value for studying ICP biosynthesis.

## Materials and methods

### Isolation and 16S rRNA gene sequencing

The new strain BtX023 (CCTCC M 2018283) was isolated based on the method promoted by Travers [25]. Soil samples were collected from a forest close to an artificial lake in Xiangtan, Hunan Province, China. Every sample was diluted into 0.1 g/mL, incubated on BP medium (per liter: 3 g beef extract, 5 g tryptone, and 5 g NaCl) added with 0.4 M sodium acetate for 4 h at 30 °C, and then heat shocked at 65 °C for 5 min.

The total genome of BtX023 was extracted with Bacterial DNA Kit (OMEGA, USA) after cultured in LB medium (per liter: 10 g tryptone, 5 g yeast extract, and 10 g NaCl). The 16S rRNA gene was amplified by the universal primer (F: AGAGTTTGATCCTGGCTCAG and R: GGTTACCTTGTTACGACTT) [26] and purified with DNA Purification Kit (BioTeke Corporation, Beijing, China). The purified 16S sequence was linked to pMD18-T Vector (TaKaRa, Japan) and transferred into *Escherichia coli* DH5α (our laboratory). The positive clone of recombinant plasmid was screened by the solid LB plate containing ampicillin (100 μg/mL), and it was sent to Sangon Biotech (Shanghai) for sequencing.

### Electron microscopy sample preparation

The strain was inoculated into LB medium overnight, and then with 1% transferred to a fermentation medium (18 g/L glucose, 14.5 g/L tryptone, 2.5 g/L KH_2_PO_4_·3H_2_O, 0.02 g/L FeSO_4_·7H_2_O, 0.02 g/L MnSO_4_·H_2_O, and 0.25 g/L MgSO_4_·7H_2_O). After fermented 24 h, 36 h and 60 h, 9,000 g for 5 min, the sediment washed 10 times with PBS buffer (20 mM), fixed overnight with 2.5% glutaraldehyde solution, and finally dehydrated using 30%, 50%, 70%, 80%, 90%, and 100% ethanol orderly. They were finally dropped on the coverslips for SEM (Hitachi su8010, Japan).

### Bioassay of toxicity against *Helicoverpa armigera* and *Plutella xylostella*

BtX023 was cultured in LB medium for 12 h and then inoculated (1%) into three different fermentation media, namely, CK (control without added Cu^2+^), CU medium (with Cu^2+^ at a final concentration of 1 × 10^–5^ M). After growth 60 h, the fermentation products were collected and diluted to 1.25, 2.5, 5, 10 and 20(μL/mL) mixed well with the artificial foods for the larvae of *H. armigera* and *P. xylostella*. The larvaes were cultured in three 24-well cell plates (one larva per well), with three replicates. All plates were sealed and incubated in a dark chamber at 28 ± 1 °C [16]. Finally, LC_50_ (50% lethal concentrations) was calculated with SPSS software (IBM SPSS Statistics 20) after 48 h. Non-overlapping 95% confidence intervals of LC_50_ were used as the criteria to determine significant difference in toxicities [27].

### Growth curve of BtX023

BtX023 inoculated into the CK, CU and NI fermentation media (30 mL per 300 mL flask). All samples were taken every 2 h for OD_600_ determination and then diluted tenfold or 30-fold so that the OD value was between 0.2 and 0.8 (three biological repeats). The growth observation performed every 6 h via phase-contrast microscope (ZEISS, Germany).

### Total protein extraction and quantification

Based on the growth curve of BtX023 the 36 h time point was selected in which spores were fully formed and crystal proteins produced. The cells were collected and washed with PBS buffer (10 mM, pH 7.8). They were treated with 300 μL of cell lysate buffer 0.5 M pH 8.0 Tris–HCl, 8 M urea (Sigma-Aldrich, USA), 65 mM CHAPS (Sigma-Aldrich, USA), 75 mM NaCl, 2 M thiourea, 10 μL of protease inhibitor (Sigma-Aldrich, USA), and 5 μL of PMSF. Ultrasonic crushing treatment was performed to extract the protein with the cells abundantly disrupted. The supernatant was quantified by BCA protein assay, and SDS-PAGE analyzed with 10 μg cell total protein per sample, the remaining samples were stored at − 80 °C [28].

### LFQ-MS analyzing

Protein samples were added to four volumes of acetone. The protein precipitate was preserved and then dissolved in 8 M urea buffer (pH 8.5, 100 mM Tris–HCl). Then, 10 mM trichloroethyl phosphate and 20 mM iodoacetamide were reacted with the protein precipitate for half an hour to allow denaturation and alkylation. Subsequently, the urea concentration in the sample was diluted to 1 M and subjected to enzymatic hydrolysis. These enzymatic fragments were separated and desalted by 2D-HPLC equipped with a strong cation-exchange column (BioBasic SCX; 0.32 mm × 100 mm, 5 μm) and a reversed-phase column (BioBasic-C18; 0.1 mm × 150 mm, 5 μm) and then analyzed by LC–MS/MS using the Thermo Q-Exactive Plus (ThermoFisher, San Jose, CA, USA) equipped with an ultra-high performance liquid chromatography unit (Thermo Scientific Dionex Ultimate 3000) and a Nanospray Flex Ion-Source (Thermo Scientific) [16, 20, 28–30]. After the original mass spectrometry data were converted into the mgf format file by the corresponding tools, the Maxquant software (according to the Bt protein library) was used to search for the identification and quantitative information extraction of the corresponding database. Significantly different proteins were screened using metaX software. Finally, conduct the GO, KEGG Pathway, eggNOG bioinformatics analysis.

### Real-time quantitative RT-PCR verification

After screening out differentially expressed proteins, a two-step real-time RT-PCR analysis with ABI 7500 Real-Time PCR System (Applied Biosystems, USA) using Power SYBR® Green PCR Master Mix (Applied Biosystems) was performed as previously described [29]. The primers (Additional file 4) designed by Primer version 5.0 (Premier Biosoft International, USA). The total RNAs were isolated using TRIzol™ Reagent (Invitrogen). The quality and the integrity of the RNA samples were evaluated by absorbance measured with NanoDrop 2000 (Thermo Scientific, USA) and agarose electrophoresis. DNase I was used to remove DNA genomic, and then the Revert Aid™ First Strand cDNA Synthesis Kit (Fermentas) was used to reverse transcribe the 1 μg total RNA to cDNA in accordance with the manufacturer’s instructions. The cDNAs were used as templates to perform relative qRT-PCR with 16S rRNA as the endogenous control. mRNA abundance was considered significantly up- or downregulated with the p value < 0.05 (Student's t test) [16]. The relative quantification method (delta–delta threshold cycle) was used to evaluate quantitative variation between samples examined.

## Supplementary information

**Additional file 1: Figure S1**. KEGG analysis of the proteomics. After obtaining the KEGG annotation for each protein, Pathway classification statistics were performed on the proteins, and the Pathway distribution of protein participation was identified; **Figure S2**. EggNOG annotation. EggNOG statistics were performed on the protein, and the orthologous group of the protein was identified; **Figure S3**. GO annotation of the proteomic. GO function classification statistics of the protein were obtained, and the functional distribution characteristics of the protein were identified; **Figure S4.** Map of protein molecular weight distribution. Most of the proteins it contains are concentrated in 10 kDa to 60 kDa.

**Additional file 2: Fig. S3.** ICPs identified by the proteomics.

**Additional file 3: Fig. S3.** Differential proteome which CK compared to CU.

**Additional file 4: Fig. S3.** the primers for qRT-PCR.

## Data Availability

The datasets used and/or analyzed during the current study are included in this article and its supplementary information files.
