# Supplementary material for: Enhancing the insecticidal activity of new Bacillus thuringiensis X023 by copper ions
Source: Microb Cell Fact. 2020 Oct 17;19:195. doi: 10.1186/s12934-020-01452-8 (PMC7568400; doi:10.1186/s12934-020-01452-8)
Supplement: Supplementary file 1 — Additional file 1: Figure S1. KEGG analysis of the proteomics. After obtaining the KEGG annotation for each protein, Pathway classification statistics were performed on the proteins, and the Pathway distribution of protein participation was identified; Figure S2. EggNOG annotation. EggNOG statistics were performed on the protein, and the orthologous group of the protein was identified; Figure S3. GO annotation of the proteomic. GO function classification statistics of the protein were obtained, and the functional distribution characteristics of the protein were identified; Figure S4. Map of protein molecular weight distribution. Most of the proteins it contains are concentrated in 10 kDa to 60 kDa. [file 12934_2020_1452_MOESM1_ESM.docx]

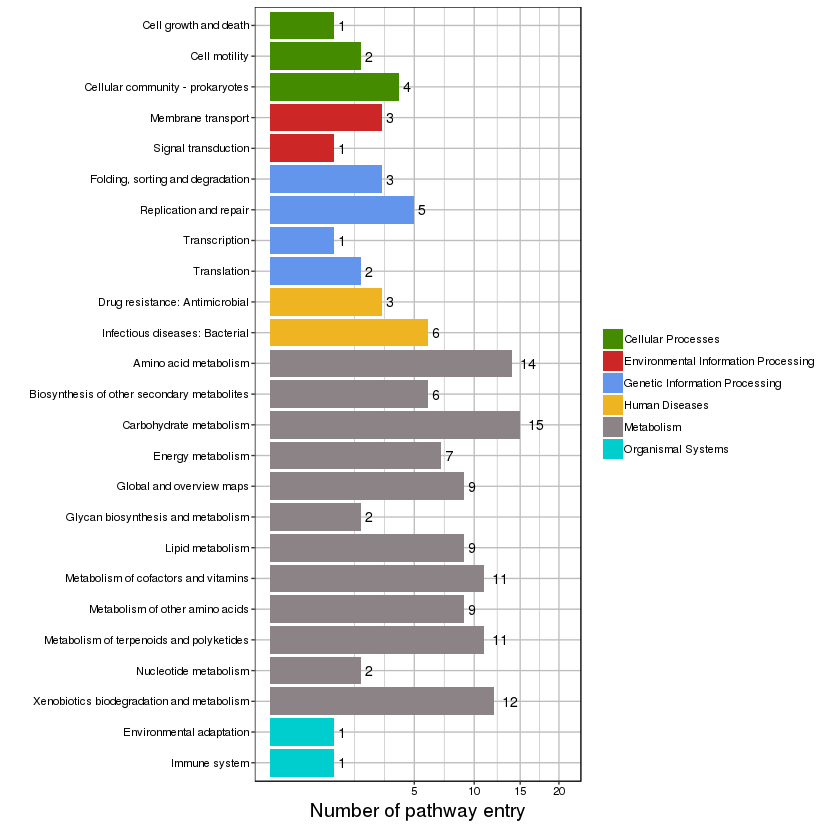


Figure S1. KEGG annotation of the proteome.


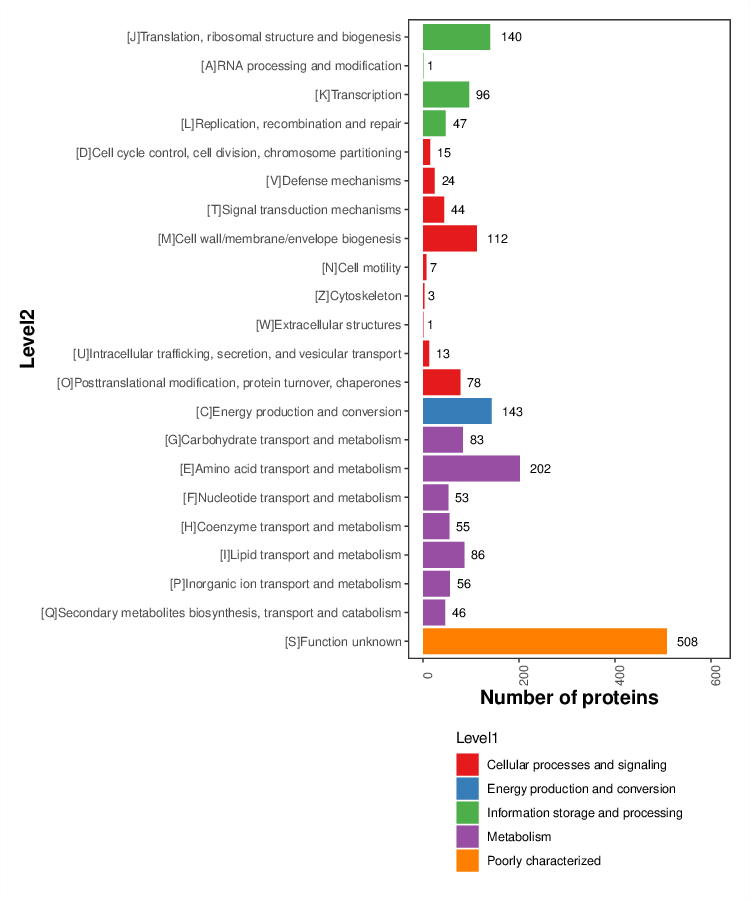


Figure S2. EggNOG annotation of the proteome.


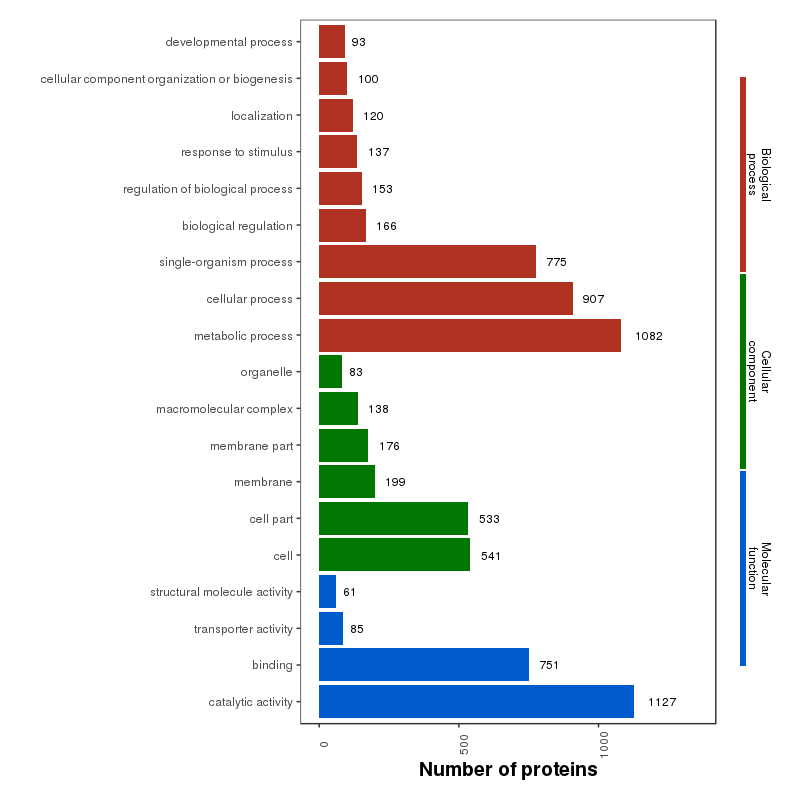


Figure S3. GO annotation of the proteome.


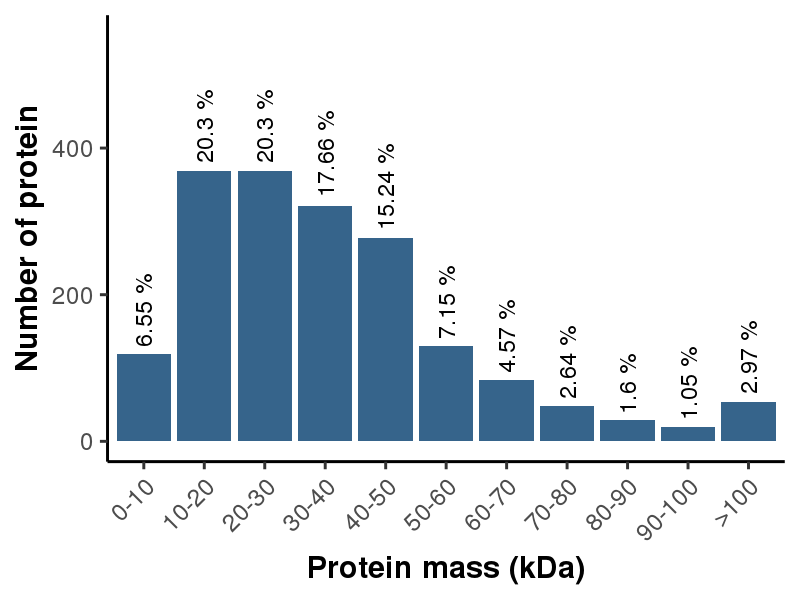


Figure S4. Protein molecular weight distribution map. Most of the proteins it contains are concentrated in 10 kDa to 60 kDa.
